# Survival analysis and mortality predictors of COVID-19 in Moroccan children: A multicenter study

**DOI:** 10.1016/j.ijregi.2026.100895

**Published:** 2026-04-11

**Authors:** Souad Yakini, Omayma Benbrik, Laila Lahlou, Mounia Amane, Maryama Bou-Iselmane, Hanane El Ghazouani, Hayat Iziki, Rachida Boutiti, El Arbi Bouaiti, Amina Barkat

**Affiliations:** 1 Laboratory of Clinical Epidemiology and Medical-Surgical Sciences, Research Team on Maternal and Child Health and Nutrition, Faculty of Medicine and Pharmacy, Mohammed V University, Rabat, 10100, Morocco; 2 Laboratory of Research and Innovation in Health Sciences, Faculty of Medicine and Pharmacy, Ibn Zohr University, Agadir, 80000, Morocco; 3 Laboratory of Health and science, Therapeutic innovation, Translational research and epidemiology, Faculty of Medicine and Pharmacy, Ibn Zohr University, Agadir, 80000, Morocco; 4 Higher institute of Nursing Professions and Health Techniques of Marrakech, Marrakech, 40000, Morocco; 5 Laboratory of Anthropology and Human Ecology, Faculty of Sciences Semlalia, Cadi Ayyad University, Marrakech, 40000, Morocco; 6 Laboratory of Biostatistics, Clinical Research, and Epidemiology, Faculty of Medicine and Pharmacy, Mohammed V University, Rabat, 10100, Morocco

**Keywords:** Newborn, Child, Predictive factors, COVID-19, Mortality, survival analysis, Morocco

## Abstract

**Objectives:**

Although COVID-19 in children is benign, it has revealed a critical vulnerability in the neonatal population. This study aimed to identify the mortality predictors and determinants of survival in children affected by COVID-19 in the Marrakech-Safi (RMS) region of Morocco.

**Methods:**

This was a retrospective, multicenter cohort study conducted among children hospitalized with COVID-19 between March 2020 and December 2021 in two RMS hospitals. All patients aged under 15 years with confirmed SARS-CoV-2 infection were included; incomplete files, misdiagnoses, and transferred patients were excluded. Overall survival was assessed using the Kaplan–Meier curve, associations were examined with the log-rank test, and predictors were identified using Cox proportional hazards regression models.

**Results:**

A total of 167 children were identified, including 52.7% females and 20.4% (n = 34) neonates. The death rate was 4.79%, with a mean overall survival of 40.9 days. The analysis identified thrombocytopenia (adjusted hazard ratio [aHR] = 87.07, 95% confidence interval [CI]: 59.85-126.77, *P* <0.001), cardiopathy (aHR = 34.95, 95% CI: 3.41-358.23, *P* = 0.003), maternal-fetal infection (aHR = 57.65, 95% CI : 4.00-831.62, *P* = 0.003), and the presence of respiratory distress (aHR = 48.37, 95% CI: 3.30-709.01, *P* = 0.005) as major mortality predictors.

**Conclusion:**

Implementing rapid intervention and preventive medicine strategies is essential to reducing morbidity and mortality in pediatric populations during future public health emergencies.

## Highlights

• A 2-year multicenter cohort of hospitalized pediatric COVID-19 in Morocco.
• In-hospital mortality reached 4.79%.
• Neonatal age, thrombocytopenia, and cardiopathy predicted mortality.
• Respiratory distress and maternal-fetal infection increased risk of death.
• Findings highlight key prognostic factors to guide timely interventions.

## Graphical Abstract

**Figure:**
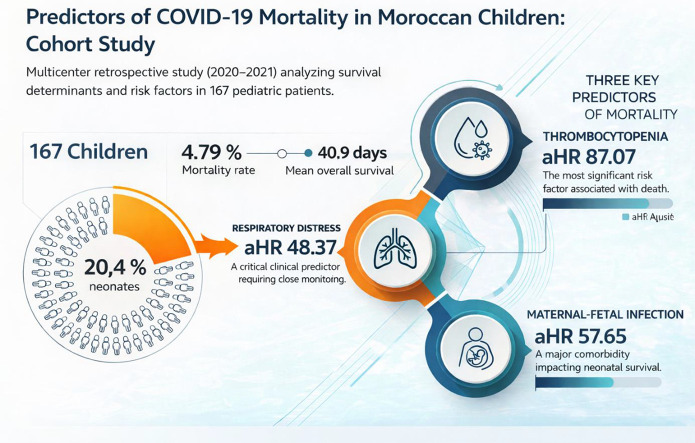

## Introduction

COVID-19 is a viral disease caused by SARS-CoV-2, a coronavirus closely related to SARS-CoV-1. Transmitted mainly by droplets and aerosols emitted by symptomatic or asymptomatic individuals, the virus replicates in the respiratory tract and can cause severe forms of infection. Its emergence at the end of 2019 profoundly disrupted health systems, economies, and social structures on a global scale, leading to the declaration of a public health emergency of international concern [[1], [2], [3]].

All age groups were susceptible to infection; however, children accounted for a small proportion of confirmed cases (approximately 2%) and were often asymptomatic or presented with mild symptoms [[4], [5], [6], [7]]. Severe cases predominantly occurred in children with underlying comorbidities, including cardiac disease, obesity, diabetes, asthma, or immunodeficiency [5,8,9], as well as in infants and adolescents, compared with other age groups, with a threefold higher risk and more frequent admissions to intensive care units [4,7,[9], [10], [11]].

Pediatric complications of SARS-CoV-2 infection were primarily characterized by multisystem inflammatory syndrome in children, pediatric long COVID, acute respiratory distress syndrome, pneumonia, and multiorgan failure [[12], [13], [14]].

However, worldwide, children experienced fewer severe forms of the disease than adults and demonstrated a very low case fatality rate, representing 1.8% of cases and 0.1% of deaths among children under 5 years of age, and 6.3% of cases and 0.1% of deaths among those aged 5-14 years [10]. Nevertheless, children played a significant role in community transmission, which was often silent, thereby complicating diagnostic and treatment efforts and representing a major challenge for pandemic control [10,11].

In Morocco, COVID-19 infections in children have steadily increased since the first pediatric case was reported on March 2, 2020. As of November 2021, a total of 50,344 cases had been documented among children [1,5]. At the national level, the cumulative incidence was 4.8 per 100,000 children, with more than 80% of cases occurring in the five regions most affected by the pandemic: Casablanca-Settat, Drâa-Tafilalet, Marrakech-Safi, Fès-Meknès, and Tanger-Tétouan-Al Hoceima [1,5].

The COVID-19 pandemic has had multiple repercussions on child health. The infection has exhibited clinical heterogeneity, with most children remaining unaffected, whereas a minority developed severe disease [5]. Conversely, the pandemic has significantly disrupted essential health services for children, including interruptions in vaccination programs and reduced access to maternal and child health services [15,16].

Therefore, the scarcity of precise data on SARS-CoV-2 infection among children in the Marrakech-Safi Region, Morocco underscores the need to fill this knowledge gap. Accordingly, this study aims to determine prognostic factors associated with survival in children with COVID-19 in Morocco.

## Patients and Methods

### Study design and setting

This was a retrospective multicenter cohort study conducted at two hospital centers in the Moroccan region: the University Hospital Center (CHU) Mohammed VI and the Regional Hospital Center (CHR) Ibne Zohr. These sites were selected because they served as the two main referral centers for the management of all pediatric COVID-19 cases during the pandemic from the RMS region and, occasionally, from other southern regions of Morocco. The study covered the period from March 9, 2020 to December 31, 2021.

### Study population

*A priori*, the sample size estimation was performed based on the primary outcome of in-hospital mortality due to COVID-19. An expected prevalence of 4% was assumed based on data from a large cohort reporting an overall in-hospital COVID-19 mortality rate in low- and middle-income countries of 4.0% (95% confidence interval [CI]: 3.6-4.4) [17]. Using a 95% confidence level and a precision of 5%, the minimum required sample size was estimated at 60 participants. This number was increased by 10% to improve the precision of the estimates, resulting in a target sample size of 66 participants [18]. To maximize statistical power and minimize selection bias, all eligible pediatric patients hospitalized with laboratory-confirmed COVID-19 (polymerase chain reaction [PCR] or antigen testing) in the two participating centers during the study period were consecutively included, based on hospital admission and mortality registries, thereby providing an exhaustive sample for this setting and period.

### Inclusion and exclusion criteria

All records of children meeting the inclusion criteria were included: children aged 0-15 years, with a confirmed diagnosis of COVID-19 based on a positive PCR or antigen test.

Exclusion criteria were as follows: records of children initially diagnosed with COVID-19 but subsequently found to have another confirmed diagnosis, records of children transferred to other facilities without available initial clinical data, incomplete or unavailable medical records, and records previously used in the pretest of the data collection tool.

### Data collection

Data were obtained from hospital registries, death registers, and medical records of patients hospitalized for COVID-19 in the Mother-Child Department of CHU Mohammed VI, as well as in the departments of CHR Marrakech.

Data collection was conducted using a standardized data extraction form, developed based on a review of the existing scientific literature and the objectives of the study [[4], [5], [6], [7],[9], [10], [11], [12], [13], [14]]. Before its use, the form was pre-tested on a sample of records to ensure its clarity and the relevance of the included variables.

The form was organized into the following sections, specifying the variables collected:

• Section 1: Sociodemographic and exposure variables: age, sex, socio-economic status, household composition, and details of potential exposure to SARS-CoV-2.
• Section 2: Clinical and laboratory variables:
  ○ Medical history: preexisting conditions, previous hospitalizations.
  ○ Clinical signs: respiratory, neurological, cardiovascular, and digestive symptoms.
  ○ Paraclinical results: SARS-CoV-2 PCR, C-reactive protein, complete blood count, chest imaging findings.
  ○ Therapeutic interventions: oxygen therapy, ventilation, medications administered.
• Section 3: Follow-up and outcome variables: length of hospital stays, duration of study participation, occurrence of complications, and patient status at discharge (recovery or death).

**Figure 1:**
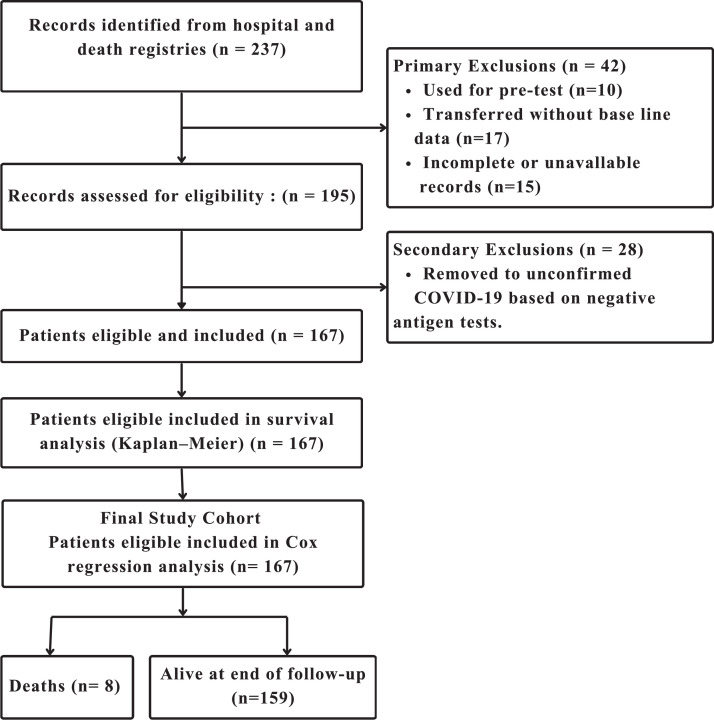
Flow diagram of Moroccan pediatric patients with SARS-CoV-2 included in the study cohort during the 2020-2021 pandemic. This flow diagram illustrates the selection of Moroccan children hospitalized with SARS-CoV-2 during the 2020-2021 pandemic. It shows the numbers of patients assessed for eligibility, excluded, and included in the study cohort, along with outcomes, including the number of deaths, in accordance with STROBE guidelines for observational studies.

Records with missing key variables or that were unavailable were excluded from all analyses (available-case analysis), and the flow diagram (Figure 1) clearly shows the number of records excluded for this reason, enhancing transparency of participant selection. Given the relatively low number of missing records, the impact on the results is expected to be minimal, and this approach was considered appropriate and methodologically sound. To further minimize potential selection and information bias, strict inclusion and exclusion criteria were applied, and data collection was conducted by personnel trained in study procedures and research ethics. Multiple quality control measures, including standardized data entry, verification, and cross-checking, were implemented to ensure the accuracy, consistency, and reliability of the data set.

### Outcome measure

The primary outcome in this study was death of the child or newborn with COVID-19 occurring during hospitalization, regardless of the cause.

### Statistical analysis

Data were entered, coded, and analyzed using Jamovi 26 statistical software. Descriptive analysis was performed to characterize the study population. Qualitative variables were presented as counts and percentages and quantitative variables as mean ± SD or median and interquartile range, depending on data distribution. Survival analysis was conducted to evaluate overall survival of children with COVID-19. Time to the primary event (death) was calculated in days from the date of hospital admission to the date of death or last follow-up for censored cases (patients discharged alive or lost to follow-up). Survival curves were generated using the Kaplan–Meier method to estimate cumulative survival probability over time. Comparisons between subgroups (age groups, comorbidity status, type of COVID-19 involvement, laboratory results) were performed using the log-rank test. Furthermore, multivariate analysis using Cox proportional hazards regression was conducted to identify variables independently associated with the risk of death. Variables with a *P* <0.3 in univariate analysis or considered clinically relevant were included in the model. Results were expressed as adjusted hazard ratios (aHRs) with 95% CIs. Statistical significance was set at *P* <0.05.

## Results

### Population characteristics

A total of 237 records were identified from hospital and death registries between March 2020 and December 2021. After several exclusions, including 28 cases of unconfirmed initial COVID-19 diagnosis, 17 records transferred to another facility without base line data, and 15 incomplete or unavailable records (3.4%), 167 pediatric participants were included and analyzed; of these, 159 survived during the follow-up period (Figure 1).

Among the 167 children hospitalized with COVID-19 during the pandemic period, 52.7% children (n = 88) were female. The median age was 60 months (interquartile range: 0.032-180 months), with the age group from 28 days to 5 years being the most represented (31.2%, n = 52).

Clinically, 63.8% of children had clinical signs on admission. These were predominantly respiratory (35.3%), digestive (25.7%), and cardiocirculatory (25.1%). The median length of hospital stay was 8 days, with an interquartile range of 3-11 days (Table 1). Over the study period, eight deaths were recorded, corresponding to death rate of 4.79% (2.33-9.36).

**Table 1:** Distribution of demographic and clinical characteristics among pediatric and neonatal patients with COVID-19.

Variables		Total n (%)	Frequency = n (%)		*P*-value
		(N = 167)	Death 8 (4.79)	Survivor 159 (95.2)	
Provenance	Urban	144 (13.8)	7 (4.2)	137 (82.0)	0.91
	Rural	23 (86.2)	1 (0.6)	22 (13.2)	
Place of admission	CHU	111 (33.5)	7 (4.2)	104 (62.3)	0.19
	CHR	56 (66.5)	1 (0.6)	55 (32.9)	
Sex	Female	88 (52.7)	2 (1.2)	86 (51.5)	0.1
	Male	79 (47.3)	6 (3.6)	73 (43.7)	
Age	———-	60 (6;120)^a^	0.346 (0.0320;12.6)^a^	72 (8;120)^a^	
Age range	Neonates	34 (20.4)	6 (3.6)	28 (16.8)	0.003
	28 days-5 years	52 (31.2)	1 (0.6)	51 (30.6)	
	6-10 years	46 (27.5)	0 (0.0)	46 (27.5)	
	11-15 years	35 (21.0)	1 (0.6)	34 (20.4)	
Comorbidity	Yes	38 (22.8)	7 (4.2)	31 (18.6)	<0.001
	No	129 (77.2)	1 (0.6)	128 (76.6)	
Prematurity	No	151 (90.4)	7 (4.2)	144 (86.2)	0.56
	Yes	16 (9.6)	1 (0.6)	15 (9.0)	
Neonatal respiratory distress	No	145 (86.8)	2(1.2)	143 (85.6)	<0.001
	Yes	22 (13.2)	6(3.6)	16 (9.6)	
Maternal-fetal infection	No	164 (98.2)	7 (4.2)	157 (94.0)	0.019
	Yes	3 (1.8)	1 (0.6)	2 (1.2)	
Heart disease	No	164 (98.2)	7 (4.2)	157 (94.0)	0.047
	Yes	3 (1.8)	1 (0.6)	2 (1.2)	
Thrombocytopenia	No	166 (99.4)	7 (4.2)	159 (95.2)	0.048
	Yes	1 (0.6)	1 (0.6)	0 (0)	
Symptomatology	No	50 (36.2)	1 (0.7)	49 (35.5)	0.150
	Yes	88 (63.8)	7 (5.1)	81 (58.7)	
Symptoms otorhino laryngeal (ENT)	Yes	21 (12.6)	6 (3.6)	53 (31.7)	0.99
	No	146 (87.4)	2 (1.2)	106 (63.5)	
Respiratory symptoms	Yes	59 (35.3)	6 (3.6)	53 (31.7)	0.016
	No	108 (64.7)	2 (1.2)	106 (63.7)	
Cardiocirculatory symptoms	Yes	42 (25.1)	8 (4.8)	34 (20.4)	<0.001
	No	125 (74.9)	0 (0.0)	125 (74.9)	
Digestive symptoms	Yes	43 (25.7)	3 (1.8)	40 (24.0)	0.43
	No	124 (74.3)	5 (3.0)	119 (71.3)	
Neurosensory symptoms	Yes	26 (15.6)	2 (1.2)	24 (1.2)	0.45
	No	141 (84.4)	6 (3.6)	135 (80.8)	
Respiratory distress	No	144 (86.2)	3 (1.8)	141 (84.4)	0.011
	Yes	23 (13.8)	5 (3.0)	18 (10.8)	
Dyspnea	No	141 (84.4)	2 (1.2)	139 (83.2)	<0.001
	Yes	26 (15.6)	6 (3.6)	20 (12.0)	
Skin mottling	No	158 (95.2)	4 (2.4)	154 (92.8)	0.002
	Yes	8 (4.8)	3 (3.0)	5 (3.0)	
Diarrhea	No	145 (87.3)	4 (2.4)	141 (84.9)	0.044
	Yes	21 (12.7)	3 (1.8)	18 (10.8)	
Thrombocytopenia	No	166 (99.4)	7 (4.2)	159 (95.2)	0.048
	Yes	1 (0.6)	1 (0.6)	0 (0)	
Average length of stay	———	8 (3;11)^a^	8 (3.5;10.25)^a^	8 (2.5;11.0)^a^	0.91

a Expressed as median and interquartile range.

Table 1 presents a detailed overview of demographic and clinical characteristics among pediatric and neonatal patients with COVID-19.

### Survival analysis

**Figure 2:**
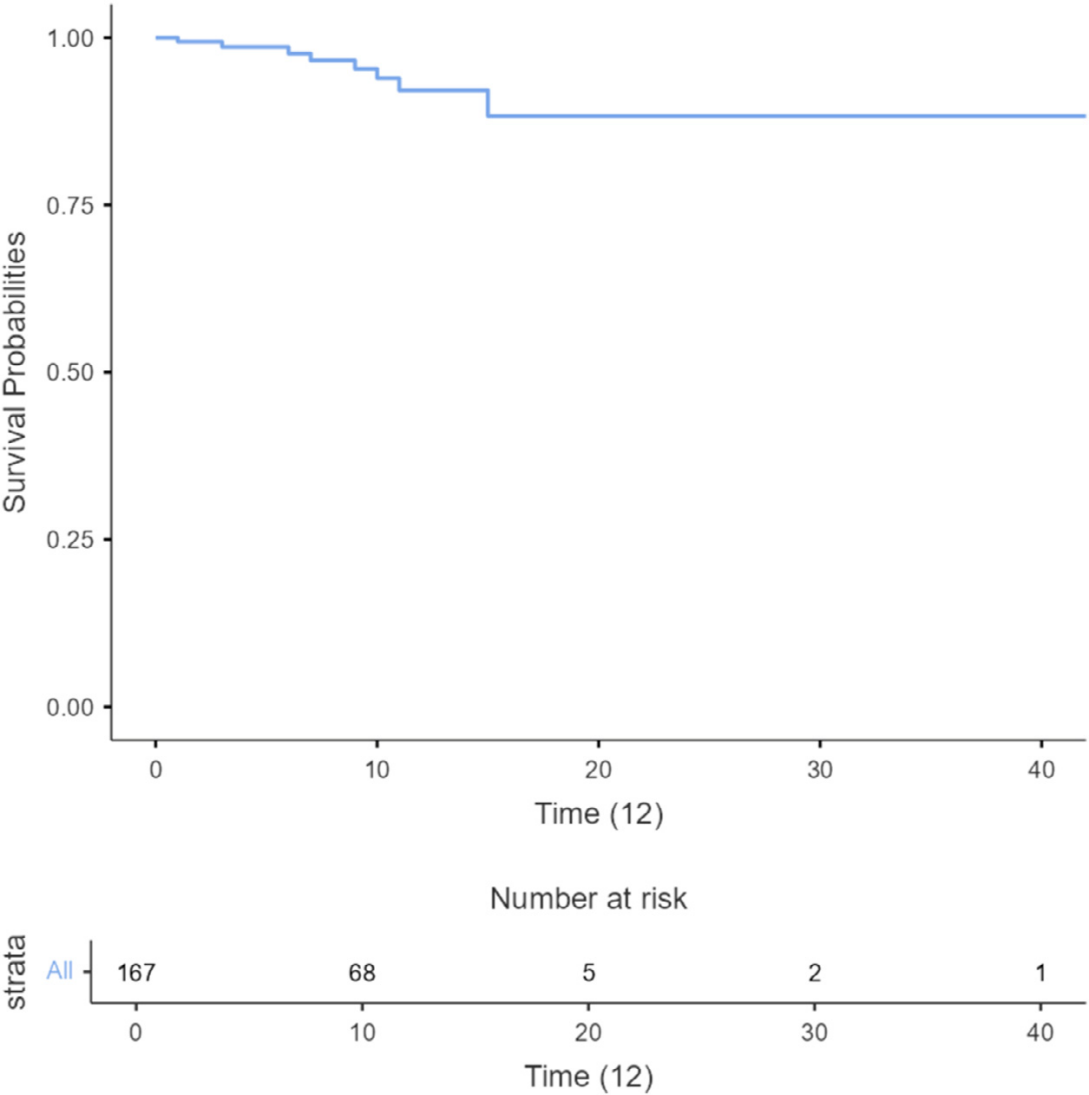
Overall survival curve for all children hospitalized with COVID-19 during the pandemic period 2020-2021. Overall survival curve for all children hospitalized with COVID-19 during the pandemic period. According to the Kaplan–Meier curve, the mean overall survival of our population was 40.9 days.

According to the Kaplan–Meier curve, the mean overall survival of our population was 40.9 days (Figure 2).

**Figure 3:**
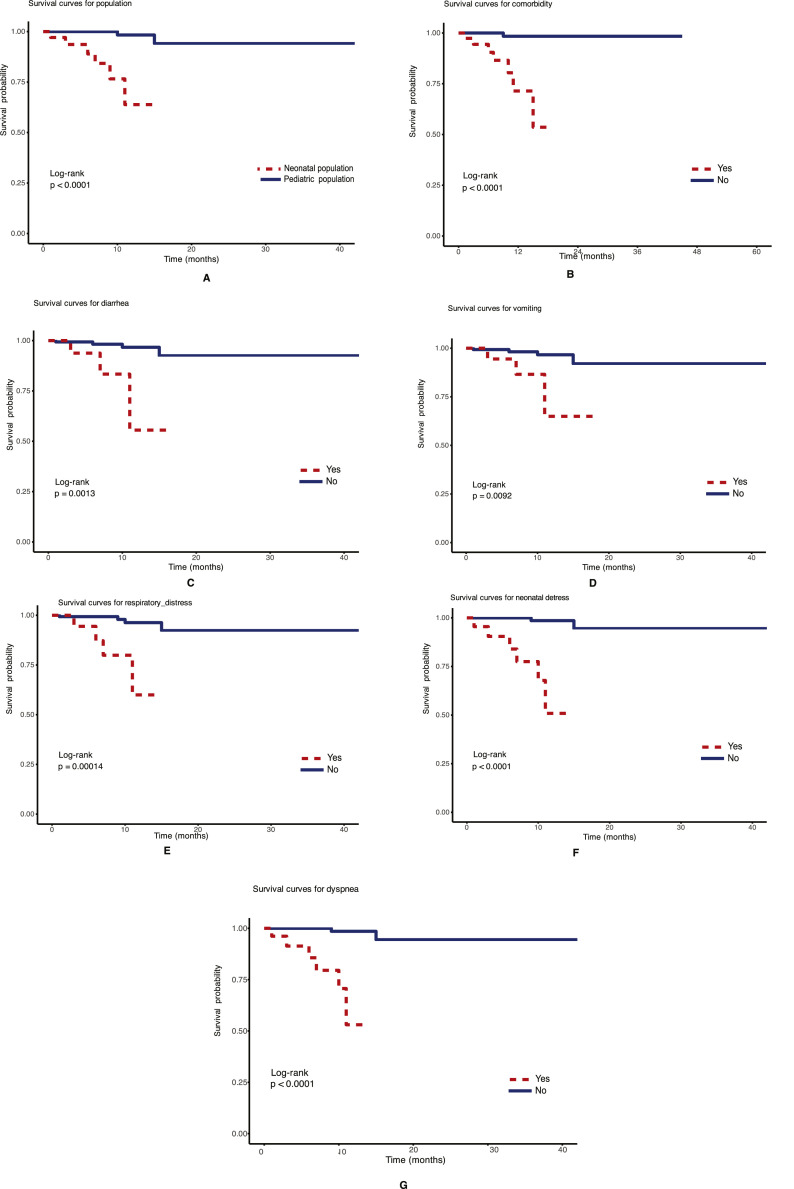
Survival curves for factors statistically significant with mortality in children hospitalized for COVID-19 during the pandemic period 2020-2021. (a) Neonatal population. (b) Comorbidities. (c) Diarrhea. (d) Vomiting. (e) Respiratory distress. (f) Neonatal respiratory distress. (g) Dyspnea. This figure shows the survival curves of children hospitalized for COVID-19 according to factors significantly associated with mortality. Panels a-g illustrate the effects of neonatal population, comorbidities, diarrhea, vomiting, general respiratory distress, neonatal respiratory distress, and dyspnea on decreased survival.

The univariate analysis identified several factors associated with survival in children hospitalized for COVID-19 during the pandemic period (*P* <0.3). The main poor prognostic factors were neonatal respiratory distress (hazard ratio [HR] = 44.80, 95% CI: 5.35-375.42, *P* <0.001), asthma (HR = 101.55, 95% CI: 6.26-164.02, *P* = 0.001), and dyspnea (HR = 40.44, 95% CI: 4.81-340.1, *P* = 0.001). Detailed results of this univariate analysis are presented in Table 2 and Figure 3.

**Table 2:** Univariate analysis of factors predicting mortality in children hospitalized for COVID-19 during the pandemic period (2020/2021).

Variables	Hazard ratio (95% confidence interval)	*P*-value
Neonatal population	19.08 [3.60-100.99]	0.001
Neonatal respiratory distress	44.80 [5.35-375.42]	<0.001
Asthma	101.55 [6.26-164.02]	0.001
Dyspnea	40.44 [4.81-340.11]	0.001
Sibilant rales	15.57 [1.73-140.45]	0.014
Respiratory distress	10.56 [2.34-47.70]	0.002
Skin mottling	14.32 [3.18-64.42]	0.001
Comorbidity	26.36 [3.23-215.24]	0.002
Diarrhea	8.36 [1.81-38.63]	0.007
Vomiting	5.89 [1.30-26.70]	0.021
Heart disease	11.55 [1.34-99.28]	0,026
Thrombocytopenia	9.63 [1.13-82.29]	0.039
Maternal-fetal infection	8.86 [1.06-73.98]	0.044

Multivariate Cox regression analysis identified the independent predictors of mortality: thrombocytopenia (aHR = 87.07, 95% CI: 59.85-126.77, *P* <0.001), heart disease (aHR = 34.95, 95% CI: 3.41-358.23, *P* = 0.003), neonatal infection (aHR = 57.65, 95% CI: 4.00-831.62, *P* = 0.003), and presence of respiratory distress (aHR = 48.37, 95% CI: 3.30-709.01, *P* = 0.005) (Table 3).

**Table 3:** Multivariate analysis of factors predicting mortality in children hospitalized for COVID-19 during the pandemic period (Cox model).

Predictive factors	Terms and conditions	Frequency n (%)	Adjusted hazard ratio (95% confidence interval)	*P*-value
		N = 167		
Thrombocytopenia	Yes	1 (0.6)	87.07 [59.85-126.77]	<0.001
	No	165 (99.4)		
Heart disease	Yes	3 (1.8)	34.95 [3.41-358.23]	0.003
	No	163 (98.2)		
Maternal-fetal infection	Yes	3 (1.8)	57.65 [4.00-831.62]	0.003
	No	163 (98.2)		
Respiratory dyspnea	Yes	26 (15.7)	48.37 [3.30-709.01]	0.005
	No	140 (84.3)		

## Discussion

Given the substantial impact of SARS-CoV-2 on pediatric morbidity and mortality, to the best of our knowledge, this multicenter study constitutes the first regional analysis to investigate prognostic factors for survival in this population. Among the 167 cases analyzed, the death rate was 4.79%. Cox regression analysis identified four major independent predictors of mortality in pediatric and neonatal patients: maternal-fetal infection, congenital heart disease, respiratory distress, and thrombocytopenia. Furthermore, the mean overall survival in critical cases was 40.9 days, reflecting the rapid progression to death and underscoring the vital importance of early identification of risk factors to guide timely therapeutic escalation.

The overall case fatality rate of 4.79% in our cohort markedly deviates from the low pediatric mortality reported worldwide (0.4%) [20], as well as the rates reported in some studies, ranging from 0.08% to 2% [7,9,21,22]. The observed divergence could be attributed to our study population, primarily composed of critically ill hospitalized patients, inherently predisposed to unfavorable outcomes [23]. Furthermore, severe cases were drawn not only from the Marrakech prefecture but also from across the region and, at times, from other southern areas. Despite representing a minority (20.4%), neonates accounted for a disproportionate share of deaths, underscoring their increased susceptibility to COVID-19, especially in the context of maternal-fetal transmission (aHR = 57.65, 95% CI: 4.00-831.62, *P* = 0.003). According to previous studies [[24], [25], [26]], this risk may be up to five times higher than that observed in adolescents, partly explained by associated factors such as prematurity and low birth weight [[24], [25], [26]]. This pattern aligns with the persistent global context, in which infant mortality continues to be strongly influenced by the predominance of neonatal deaths (47%) [25], [27], [28]. Consequently, neonates represent a key priority for targeted health care strategies and interventions.

The cohort’s demographic profile revealed a slight female predominance (52.7%), with the highest incidence observed among children aged 28 days to 5 years. This pattern closely aligns with the study by Esteves e*t al.* that was conducted in Rio de Janeiro [9] yet contrasts with international reports, where a male predominance is generally observed [14,20,22]. Nonetheless, it is noteworthy that mortality patterns contrast with incidence: despite a higher case rate in girls, excess deaths were primarily observed in boys (75% of deaths, n = 6), reflecting the well-documented increased vulnerability of males to SARS-CoV-2 (odds ratio 2,67, CI 95%: 2.07-3.44, *P* <0.001) [1,12,28].

From a clinical perspective, this study underscores the critical impact of comorbidities, especially cardiovascular disorders, on COVID-19–related pediatric mortality [29]. In our cohort, cardiac disease emerged as a significant predictor of mortality, aligning with previous research indicating up to a 7.74-fold elevated risk among pediatric patients with cardiac disorders [1,9,26,29]. This heightened vulnerability may be attributed to the limited myocardial functional reserve and the heart’s pronounced susceptibility to the systemic inflammatory response triggered by SARS-CoV-2 (cardiomyopathy), even in previously healthy patients, positioning preexisting cardiac conditions as a key determinant of unfavorable outcomes [12]. Moreover, dyspnea was identified as a significant predictor of mortality (aHR = 48.37), highlighting the extent of pulmonary compromise. This finding aligns with existing literature, which recognizes dyspnea as a critical marker of disease severity and a powerful predictor of death in pediatric patients with COVID-19 [9,14,15,28]. From a biological standpoint, thrombocytopenia was identified as the strongest predictor of adverse outcomes (aHR = 87.07, 95% CI: 59.85-126.77), underscoring its significance as a critical alarm marker. This finding likely reflects coagulopathy or severe systemic inflammatory syndrome and aligns with existing literature, in which dyspnea is linked to severe SARS-CoV-2 complications, including death (risk ratio = 2.34, 95% CI: 1.23-4.45, *P* <0,001; I² = 96.8%) [[30], [31], [32]].

These results highlight the importance of early identification and close monitoring of high-risk children, particularly, neonates and those with cardiac or hematologic abnormalities. However, certain limitations should be considered. The retrospective and hospital-based design may introduce selection bias because severe cases are likely overrepresented, potentially overestimating mortality. In addition, some confounding factors, such as nutritional status or socio-economic level, were not fully accounted for, limiting data accuracy and preventing the establishment of other causal relationships. The relatively small number of events (n = 8) may compromise the stability of the multivariable Cox regression model and increase the risk of overfitting and instability of the estimated coefficients. In this context, the multivariable analysis was conducted for exploratory purposes, with emphasis on the direction of associations and their clinical plausibility rather than on the precision of the estimates. Therefore, these results should be interpreted with caution, and our findings should be confirmed by studies including a larger number of events. Finally, the specific health care context restricts the generalizability of the findings to other settings.

## Conclusion

Although the case fatality rate of 4.79% exceeds international references, our results reaffirm the relatively low mortality of pediatric COVID-19. Five key prognostic factors were identified: neonatal age, maternal-fetal transmission, cardiac disease, respiratory distress, and thrombocytopenia. These findings underscore the need for targeted screening and the development of context-adapted intensive care protocols that integrate clinical and biological risk indicators, with close monitoring of biological markers and respiratory status, especially in neonates. At a broader level, the study informs public health policies to optimize resource allocation and improve preparedness for future pediatric infectious disease outbreaks. The external validity is supported by the inclusion of the entire pediatric population across urban and rural settings, accounting for geographical, socio-economic, and structural diversity. Nevertheless, caution is warranted when extrapolating these findings to other healthcare contexts. The external validity of this study is supported by the representativeness of the included all pediatric population and the coverage of the entire pandemic period. By encompassing urban and rural settings and accounting for geographical, socio-economic, and structural diversity, the study provides a comprehensive view of COVID-19’s impact in the region and strengthens the generalizability of the results. Nevertheless, caution is warranted when extrapolating these findings to other health care contexts.

## Declaration of competing interest

The authors have no competing interests to declare.
